# Gastrointestinal Colonization with Vancomycin-Resistant Enterococci In Hospitalized and Outpatients

**DOI:** 10.3889/oamjms.2015.002

**Published:** 2014-11-05

**Authors:** Elena Trajkovska-Dokic, Ana Kaftandzieva, Snezana Stojkovska, Aneta Kuzmanovska, Nikola Panovski

**Affiliations:** 1 *Institute for Microbiology and Parasitology, Medical Faculty, Ss Cyril and Methodius University of Skopje, Skopje, Republic of Macedonia*; 2 *University Clinic for Infective Diseases and Febrile Conditions, Ss Cyril and Methodius University of Skopje, Skopje, Republic of Macedonia*

**Keywords:** colonization, vancomycin, resistance, Enterococcus, genotypes

## Abstract

**BACKGROUND:**

The incidence of infection and intestinal colonization with vancomycin resistant enterococci (VRE) is increasing in many countries in the last decade. Concerning the difficult antimicrobial treatment of infections caused by VRE, decreasing the incidence and prevalence of these infections is an important factor in VRE-induced morbidity and mortality control.

**AIM:**

To determine the prevalence of gastrointestinal colonization with vancomycin resistant enterococci in hospitalized and outpatients, and to determine the genetic base of the vancomycin resistance in VRE isolates.

**MATERIAL AND METHODS:**

Seven hundred and eighty stool specimens were investigated for the gastrointestinal carriage of vancomycin-resistant enterococci (VRE). Susceptibility to vancomycin was tested in all isolates by disk-diffusion test and E-test (AB Biodisk, Sweden). Determined vancomycin resistant enterococci were than tested for detection of *vanA*, *vanB* and *vanC* genes by PCR.

**RESULTS:**

Vancomycin resistant strains of *enterococci* were isolated from 46 (16.1 %) of the 285 hospitalized patients and 5 (7.7 %) of the 65 patients living in the community (p < 0.05). The most of the highly resistant enterococci strains to vancomycin (95.2 %), were identified as *E. faecium*. Minimal inhibitory concentrations (MICs) to vancomycin in all 39 *vanA* genotypes of *E. faecium* and two *vanA* genotypes of *E. fecalis* were > 256 μg/ml. Three *vanB* genotypes of *E. faecium* and one *vanB* genotype of *E. faecalis* had MICs of 32 μg/ml. All six *vanC* genotypes of *E. gallinarum* had MICs of 8 μg/ml. All *vanA* genotypes of VRE were highly resistant to vancomycin, with MICs above 256 μg/ml. Three *vanB* genotypes of VR *E. faecium* and one VR *E. fecalis* were resistant, with MICs 32 μg/ml. *vanC* genotypes of VR *E. gallinarum* were intermediate resistant to vancomycin with MICs of 8 μg/ml.

**CONCLUSIONS:**

The prevalence of vancomycin resistant enterococci in Republic of Macedonia was 2-fold higher in hospitalized than in outpatients. *VanA* genotype was dominant in isolates of *E. faecium* and it was highly associated with the MIC values above the 256 μg/ml. Since most of the enterococcal infections are endogenous, there is a need for screening the colonization of patient’s intestinal flora with VRE at the hospital entry. Identification and genotyping of faecal enterococci, together with their susceptibility testing to vancomycin, could be useful marker for the infection control.

## Introduction

Enterococci are inhabitants of the gastrointestinal tract which can colonize particular parts of the human body and cause: intra-abdominal infections, urinary tract infections, wound infections, endocarditis, bloodstream infections and sepsis [1-3]. Enterococci are quite resistant microorganisms, surviving in both animate and inanimate surfaces: bathroom sinks, thermometers, bed rails, night tables and other hospital equipment, for extended period of time [4]. This feature of enterococci increases their potential for spreading in the surrounding and for their transmition from person-to-person very easily.

With the increasing resistance of enterococci to penicillins and aminoglycosides, the addition of resistance to vancomycin severely limitate therapeutic options for those persons who develop infections with these highly resistant strains [5]. Many reports from different countries worldwide emphasize the rapid increase in the incidence of infection as well as colonization with vancomycin resistant enterococci (VRE) in the last five years [6]. Concerning the difficult antimicrobial treatment of infections caused by VRE, their decreasing is an important factor in VRE-induced morbidity and mortality control. It seems that the high rate of patient’s VRE colonization is an important factor in infection control. Many risk factors play an important role in colonization and infection with VRE: prolonged hospitalization, serious medical conditions, intensive care unit stays, abdominal and thoracic surgery, and urinary catheterization. Use of vancomycin, third generation of cephalosporins and antibiotics with anaerobic activity (imipenem, metronidazole and clindamycin) significantly associate with colonization and infection with VRE. Many studies have shown that intestinal colonization of VRE depends on geographic factors [7].

Since the prevalence of VRE as a health marker for infection control in hospitals has not been investigated in Republic of Macedonia, the objectives of the study was to determine the prevalence of gastrointestinal colonization with Vancomycin-Resistant Enterococci in hospitalized and outpatients, and to determine the genetic base of the vancomycin resistance in VRE isolates.

## Material and Methods

In a period of 3 months (August to October 2013), the Laboratory for enteric infections at the Institute for Microbiology and Parasitology, Medical Faculty of the Ss Cyril and Methodius in Skopje, R. Macedonia, has received 585 stool specimens from patients between 40 and 70 years of age, hospitalized at the internal and surgery clinics, as well as clinic for infectious diseases. For each patient age, sex and length of hospital stay were recorded. In the same period, 195 stool specimens were received from the patients living in the community, with the same range of age as hospitalized patients. All 780 specimens were received for examination of bacteria as etiologic agents of gastrointestinal infections, and for investigation of the gastrointestinal carriage of vancomycin resistant enterococci (VRE). For that purpose specimens were inoculated on 5% sheep blood agar and incubated at 36-37°C for 24 hours in an aerobic conditions. Suspected colonies of enterococci were selected and identified by morphology and biochemical tests: catalase (negative) and esculin (positive) reaction.

### Susceptibility testing

Initial susceptibility to vancomycin was tested by disk-diffusion test according to the recommendations of the National Committee for Clinical Laboratory Standards [8]. It was confirmed by E-test (AB Biodisk, Sweden). Bacterial suspensions of enterococci prepared with a turbidity-adjusted to achieve a final inoculum of 105 CFU/ml, were inoculated at Mueller-Hinton agar supplemented with 5% sheep blood. They were incubated under the same conditions as during their initial isolation. The susceptibility of isolated enterococci to vancomycin was analysed according to the following criteria: MIC < 4 mg/ml was considered as sensitive to vancomycin, MIC between 4 and 16 mg/ml was considered as intermediate resistant to vancomycin, MIC equal or above 32 mg/ml was considered as resistant to vancomycin and MIC = 256 mg/ml was considered as highly resistant to vancomycin. Data were analyzed with SPSS (Version 13) software using chi-square test.

### DNA isolation

Colonies of isolated enterococci were suspended in TEG buffer (25 mM Tris-HCl, 10 mM EDTA, 50 mM Glucose). A lysosime solution (10 mg/liter) was added and the mixture was incubated for 1 hour at 37°C. DNA was eluted with 10 mM Tris-HCl (pH=8.0).

### PCR assay for vanA, vanB and vanC genes

10 μl of DNA was added to a 90 μl PCR mixture containing 10 mM Tris-HCl (pH=8.0), 50 mM KCl, 2.5 mM MgCl2, 0.2 mM (each) the four deoxyribonucleotide triphosphates, 50 pmol of each primer from the three different primer couples (Table 1) and 1.2 U of Taq DNA polymerase.

**Table 1:** Primers and expected PCR product sizes.

Gene	Primer sequence	Product size (bp)
vanA – F	5’ GGG AAA ACG ACA ATT GC 3’	732
vanA – R	5’ GTA CAA TGC GGC CGT TA 3’	
vanB – F	5’ GTG ACA AAC CGG AGG CGA GGA 3’	433
vanB – R	5’ CGC CAT CCT CCT GCA AAA AA 3’	
vanC – F	5’ CTC CTA CGA TTC TCT TG 3’	439
vanC – R	5’ CGA GCA AGA CCT TTA AG 3’	

Amplification of DNA was performed in an Applied Biosystems thermocycler by using denaturation of DNA at 94°C for 2 minutes, followed by 30 cycles of 1 minute of denaturation at 94°C, 1 minute of primers annealing at 54°C and 1 minute of extension at 72°C. Amplicons were analyzed by UV illuminator after running electrophoresis on a 1.5 % agarose gel containing 0.5 μg/ml ethidium bromide.

## Results

One- hundred- twenty- nine (45.2 %) of the 285 hospitalized patients and 19 (29.2 %) of 65 patients living in the community carried enterococci in their gastrointestinal tract (p < 0.05). The mean age of the study participants was 53.6 (SD=25.2) years. 58.5% of all participants were male. Fifty-two (40.3 %) out of 129 enterococci isolated from hospitalized patients and 6 (31.5 %) out of 19 enterococci isolated from outpatients respectively, were identified as *Enterococus faecium* (p < 0.01) (Table 2).

**Table 2:** Distribution of *Enterococcus spp.* and *E. faecium* in hospitalized and outpatients.

Number of patients	No. and % of isolated *Enterococcus spp*.	No. and % of *Enterococcus* isolates identified as *E. faecium*
Hospitalized patients (285)	129/285 45.2 %)	52/129 (40.3 %)

Outpatients (65)	19/65 (29.2 %)	6/19 (31.5 %)

Total No. of patients (350)	148/350 (42.3 %)	58/148 (39.2 %)

Therefore, *E. faecium* was isolated from 52 (18.2 %) out of 285 hospitalized patients and 6 (9.2 %) out of 65 patients living in the community (p < 0.05) (Table 3). Seventy-seven (59.7 %) out of 129 enterococci isolated from hospitalized patients and 13 (68.4 %) out of 19 enterococci isolated from outpatients belonged to *Enterococcus fecalis and Enterococcus* *gallinarum*. *Enterococcus fecalis* was identified in 56 (43.4 %) out of 129 isolates of enterococci from hospitalized patients, and in 10 (52.6 %) out of 19 isolates of enterococci from outpatients. *Enterococcus gallinarum* was identified in 21 (16.3 %) out of 129 isolates of enterococci from hospitalized patients, and in 3 (15.8 %) out of 19 isolates of enterococci from outpatients (Table 3).

**Table 3:** Distribution of three different species of enterococci in hospitalized and outpatients.

Number of patients	*E. faecium*	*E. faecalis*	*E. gallinarum*
Hospitalized patients (285)	52/285 (18.2 %)	56/129 (43.4%)	21/129 (16.3 %)

Outpatients (65)	6/65 (9.2 %)	10/19 (52.6 %)	3/19 (15.8 %)

Total No. of patients (350)	58/350 (16.5 %)	66/148 (44.6 %)	24/148 (16.2 %)

Vancomycin resistant strains of enterococci were isolated from 46 (16.1 %) of the 285 hospitalized patients, and 5 (7.7 %) of the 65 patients living in the community (p < 0.05). Forty (86.9%), 4 (8.69 %) and 2 (4.34 %) vancomycin resistant isolates of *E. faecium, E. gallinarum* and *E. fecalis* respectively, belonged to the hospitalized patients. Two (40 %), 2 (40 %) and 1 (20 %) vancomycin resistant isolates of *E*. *faecium, E. gallinarum* and *E. fecalis*, respectively, belonged to the patients living in the community (Table 4).

**Table 4:** Three different species of Vancomycin resistant enterococci (VRE) isolated from hospitalized and outpatients.

Three different species of VRE	VRE isolated from hospitalized patients No. and (%)	VRE isolated from outpatients No. and (%)
*E. faecium*	40 (86.9 %)	2 (40 %)
*E. gallinarum*	4 (8.69 %)	2 (40 %)
*E. faecalis*	2 (4.34 %)	1 (20 %)
Total	46 (100.0 %)	5 (100 %)

According to the MICs, 97 (65.6 %) out of the 148 enterococci isolated from both hospitalized and outpatients were susceptible to vancomycin (VSE), with MIC < 4 μg/ml. Eighty-three (64.3%) out of 129 VSE isolates belonged to hospitalized patients and 14 (73.5 %) out of 19 VSE isolates belonged to outpatients. In 51 (35.4 %) out of 148 isolated enterococci was detected resistance to vancomycin (vancomycin-resistant enterococci, VRE). On the basis of MICs, VRE isolates were separated into 3 groups: 6 (11.76 %) revealed *intermediate resistance* to vancomycin (group 1), 4 (7.84 %) were *resistant* to vancomycin (group 2) and 41 (80.40%) were *highly resistant* to vancomicin (group 3) (Table 4). The most of *highly resistant* VRE (95.2 %) were identified as *E. faecium*, and the other 0.8 % belonged to *E. faecalis*. Three isolates of *resistant* VRE were *E. faecium* and only one *E*. *faecalis*. All *intermediate resistant* VRE were identified as *E. gallinarum* (Table 5).

**Table 5:** MICs in three different Vancomycin Resistant *Enterococcus* (VRE) species.

Three groups of VRE according their MICs to Vancomycin (μg/ml)	Three groups of VRE species No (%)	*E. faecium* No (%)	*E. faecalis* No (%)	*E. gallinarum* No (%)
Group 1 (4 – 16)	6 (11.76 %)	/	/	6 (100 %)
Group 2 (Above 32)	4 (7.84 %)	3 (75 %)	1 (25 %)	/
Group 3 (Above 256)	41 (80.4 %)	39 (95.2 %)	2 (0.8 %)	/

Total	51 (100 %)		51 (100 %)	

**Figure 1:**
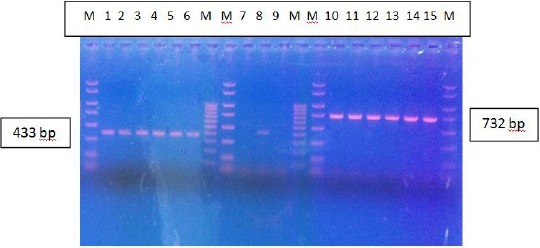
*Detection of vanA, vanB and vanC genes by PCR. (Lines: M -> PCR markers; Lines: 1, 2, 3, 4, 5, and 6 -> vanB; Line: 8 -> vanC; Lines: 10, 11, 12, 13, 14 and 15 -> vanA)*.

Molecular analysis showed the presence of three genotypes of *Enteroccocus*: *vanA, vanB* and *vanC* (Fig. 1).

Forty-one (80.4 %), 4 (7.8 %) and 6 (11.8 %) out of 51 VRE isolates has *vanA, vanB* and *vanC* genotype, respectively. *VanA* genotype was detected in 39 (92.85 %) out of 42 isolates of *Enterococcus faecium*, and 2 (66.6 %) out of 3 isolates of *Enterococcus fecalis*. *VanB* genotype was detected in 3 (7.15 %) out of 42 isolates of *E. faecium* and 1 (33.3%) out of 3 isolates of *E. fecalis*. *VanC* was detected in all 6 (100 %) isolates of *E. gallinarum* (Table 6).

**Table 6:** Three different genotypes in Vancomycin Resistant *Enterococcus* (VRE) isolates.

*van* genotypes in VRE isolates	*van* genotypes in VRE isolates No. and (%)	*E. faecium* No. and (%)	*E. faecalis* No. and (%)	*E. gallinarum* No. and (%)
*vanA*	41 (80.4)	39/42 (92.85)	2/3 (66.6)	/

*vanB*	4 (7.8)	3/42 (7.15)	1/3 (25)	/

*vanC*	6 (11.8)	/	/	6 (100)

Total number of VRE isolates	51 (100)		51 (100)	

Minimal inhibitory concentrations (MICs) to vancomycin in all 39 *vanA* genotypes of *E. faecium* and 2 *vanA* genotypes of *E. fecalis* was > 256 μg/ml. Three *vanB* genotypes of *E. faecium* and one *vanB* genotype of *E. faecalis* had MICs of 32 μg/ml. All 6 *vanC* genotypes of *E. gallinarum* had MICs of 8 μg/ml. The risk factors which are associated with VRE colonization, included prolonged length of hospital stay (12.5 days vs. 7.6 days, p = 0.001) and higher age (63.8 years vs. 48.6 years, p = 0.002).

## Discussion

The increasing incidences of colonization and infection with VRE have caused serious concern to physicians [9]. This study documents the prevalence of intestinal colonization in selected patients hospitalized at the internal clinics, surgical clinics, clinic for infective diseases, as well as patients living in the community (outpatients). Enterococci were found in 45.2 % of hospitalized patients and in 29.2 % of outpatients, and these results are in agreement with the findings in the literature [10]. *E. faecium* was isolated from 40.3 % of hospitalized and 31.5 % of outpatients, which is in agreement with previous findings of *E. faecium* [11]. In our study was not found significant difference between the prevalence of gastrointestinal tract colonization with *E. faecium* and *E. fecalis* in hospitalized patients, but the results have shown that *E. gallinarum* was significantly less common gastrointestinal tract colonizer in hospitalized patients (p < 0.05). In the group of outpatients was found the highest prevalence of gastrointestinal colonization with *E. fecalis*. The prevalence of VRE in hospitalized patients (16.1 %) was much higher than in outpatients (7.7 %) (p < 0.05). The rate of VRE colonization varies widely in different studies. The study of Gambarotto et al. showed the prevalence of VRE in hospitalized patients of 37 % [12], but in the study of Wisplinghoff et al. was shown that the prevalence of vancomycin resistant enterococci in hospitalized patients was 2 % and 60 % for *E*. *fecalis* and *E. faecium*, respectively [13]. The colonization level with VRE in outpatients from Europe parallels the colonization level of animals with this resistant microorganism [14]. We have no official data about the usage of vancomycin as a growth promoter in animals in the Republic of Macedonia, but on the bases of our findings regarding the low prevalence of VRE in outpatients with only 7.7 %, we can presume that it is not the case. n this study, we found a significant difference in the prevalence of particular species of VRE. The most prevalent species, with 86.9 % in hospitalized patients, was *E. faecium*, against the prevalence of vancomycin resistant *E. gallinarum* and vancomycin resistant *E. fecalis* with 8.69 % and 4.34 %, respectively (p < 0.05). These data agree with those found in the literature [15, 16]. Analyzing the genotypes in VRE, we found that the most prevalent genotype was *vanA* (80.4 %), against *vanB* and *vanC* genotypes, present in 7.8 % and 11.8 % of VRE isolates, respectively. *VanA* genotype was dominant in isolates of *E. faecium*, presenting 39 (92.85 %) out of 42 isolates. *VanB* genotype was detected in 3 and 1 isolate of *E. faecium* and *E. fecalis*, respectively. *VanC* was detected in all 6 isolates of *E. gallinarum*. Knowledge of the type of resistance is critical for infection control purposes. As transferable genes, *vanA* and *vanB* can spread from person to person and in that manner they can increase acquired resistance. Determination of the investigated VRE strains as *vanA* and *vanB* genotypes confirmed that the most isolates of VRE had acquired resistance. In contrast, taking in consideration that *vanC* genes are nontransferable and mediates low level of vancomycin resistance, they are not associated with serious infections and outbreaks and should not be considered important from an infection control point of view. Our findings of *vanA* and *vanB* genotypes resistancy agree with those found in the literature [17-19].

In conclusion, this study shown that the prevalence of vancomycin resistant enterococci in R. Macedonia was 2-fold higher in hospitalized than in outpatients. This high rate of VRE colonization is an important factor in infection control. *VanA* genotype was dominant in isolates of *E. faecium* and it was highly associated with the MIC values above 256 μg/ml. Since most of the enterococcal infections are endogenous, there is a need for screening the colonization of patient’s intestinal flora with VRE at the hospital entry. Identification and genotyping of fecal enterococci, together with the investigation of their susceptibility to vancomycin, could be useful marker for the infection control.

## References

[1] Murray BE (1990). The life and times of Enterococcus. Clin Microbiol Rev.

[2] Emori TG, Gaynes RP (1993). An overview of nosocomial infections, including the role of the microbiology laboratory. Clin Microbiol Rev.

[3] Schalbern DR, Culver DH, Gaynes RP (1991). Major trends in the microbial etiology of nosocomial infection. Am J Med.

[4] Grayson L, Eliopoulos GM, Wennerstern C (1991). Increasing resistance to blactam antibiotics among clinical isolates of Enterococcus faecium: a 22-year review at one institution. Antimicrob. Agents Chemother.

[5] Montecalvo MA, Horowitz H, Gerdis C (1994). Outbreak of vancomycin-ampicilin-, and aminoglycoside-resistant Enterococcus faecium bacteriemia in an adult oncology unit. Antimicrob. Agents Chemother.

[6] Handwerger S, Perlman DC (1992). Concommitant high-level vancomycin and penicillin resistance in clinical isolates of enterococci. Clin Infect Dis.

[7] Abbasali J (2008). Prevalence of vancomycin resistant enterococci colonization in hospitalized patients. Iranian Journal of Clin Infec Dis.

[8] National Committee for Clinical Laboratory Standards (1993). Methods for Dilution Antimicrobial Susceptibility Tests for Bacteria that Grow Aerobically. Approved standard M7-A3.

[9] Hospital Infections Control Practices Advisory Committee (1995). Recommendation for preventing the spread of vancomycin resistance. Am J Infect Control.

[10] Hubert P, Endtz (1997). Fecal carriage of Vancomycin-resistant Enterococci in hospitalized patients in the Netherlands. Journal of Clin Microbiol.

[11] Benno Y (2006). Comparison of the fecal microflora in rural Japaneseand urban Canadians. Microbiol Immunol.

[12] Gambarotto K, Ploy MC (2000). Prevalence of vancomycin-resistant enterococci in fecal samples from hospitalized patients and nonhospitalized controls in a cattlerearing area of France. J Clin Microbiol.

[13] Wisplinghoff H (2004). Nosocomial bloodstream infections in US hospitals: analysis of cases from a prospective study. Clin Infect Dis.

[14] Devriese L.A (1996). Presence of vancomycin-resistant enterococci in farm and pet animals. Antimicrob Agents Chemother.

[15] Mettalidis Simeon, Chatzidimitrou Maria, Tsona Afroditi (2006). Vancomycin- Resistant Enterococci, colonizing the intestinal tract of patients in a university hospital in Greece. The Brazilian Journal of Infectious Diseases.

[16] Charmaine M, Huckabee W, Charles Huskins, Patrick R. Murray (2009). Predicting clearance of colonization with Vancomycin-Resistant Enterococci and Methicillin-Resistant Staphylococcus aureus by use of weekly surveillance cultures. J Clin Microbiol.

[17] Zirakzadeh A, Patel R (2006). Vancomycin-Resistant Enterococci: colonization, infection, detection and treatment. Mayo Clin Proc.

[18] Young HL, Ballard SA, Roffey P, Grayson ML (2007). Direct detection of vanB using the Roche Light Cycler detection assay to indicate vancomycin-resistant enterococcal carriage-sensitive but not specific. J Antimicrob Chemother.

[19] Kurup A, Chlebicki MP, Ling ML (2008). Control of a hospital-wide vancomycinresistant enterococci outbreak. Am J Infect Control.

